# Mucoadhesive Microparticles for Gastroretentive Delivery: Preparation, Biodistribution and Targeting Evaluation

**DOI:** 10.3390/md12125764

**Published:** 2014-12-01

**Authors:** Jing-Yi Hou, Li-Na Gao, Fan-Yun Meng, Yuan-Lu Cui

**Affiliations:** 1 State Key Laboratory of Earth Surface Processes and Resources Ecology, Beijing Normal University, Haidian District, Beijing 100088, China; E-Mail: 201221190039@mail.bnu.edu.cn; 2 State Key Laboratory of Dao-di Herbs, China Academy of Chinese Medical Sciences, Beijing 100700, China; 3 Research Center of Traditional Chinese Medicine, Tianjin University of Traditional Chinese Medicine, Tianjin 300193, China

**Keywords:** alginate, chitosan, ethanol-induced gastric injury, mucoadhesive microparticles, puerarin

## Abstract

The aim of this research was to prepare and characterize alginate-chitosan mucoadhesive microparticles containing puerarin. The microparticles were prepared by an emulsification-internal gelatin method using a combination of chitosan and Ca^2+^ as cationic components and alginate as anions. Surface morphology, particle size, drug loading, encapsulation efficiency and swelling ratio, *in vitro* drug released, *in vitro* evaluation of mucoadhesiveness and Fluorescence imaging of the gastrointestinal tract were determined. After optimization of the formulation, the encapsulation efficiency was dramatically increased from 70.3% to 99.2%, and a highly swelling ratio was achieved with a change in particle size from 50.3 ± 11.2 μm to 124.7 ± 25.6 μm. In ethanol induced gastric ulcers, administration of puerarin mucoadhesive microparticles at doses of 150 mg/kg, 300 mg/kg, 450 mg/kg and 600 mg/kg body weight prior to ethanol ingestion significantly protected the stomach ulceration. Consequently, significant changes were observed in inflammatory cytokines, such as prostaglandin E_2_ (PGE_2_), tumor necrosis factor (TNF-α), interleukin 6 (IL-6), and interleukin1β (IL-1β), in stomach tissues compared with the ethanol control group. In conclusion, core-shell type pH-sensitive mucoadhesive microparticles loaded with puerarin could enhance puerarin bioavailability and have the potential to alleviate ethanol-mediated gastric ulcers.

## 1. Introduction

Gastric ulcers, primarily defined as damage in the gastric mucosa that penetrates through the muscularis mucosa into the submucosa [1], is a common disease, affecting an estimated 10% of the population. The basic physiopathology of gastric ulcers results from an imbalance between the defensive (mucus secretion, mucus-bicarbonate barrier, blood flow, cellular regeneration and endogenous protective agents) and aggressive (pepsin secretion, hydrochloric acid, stress and alcohol consumption) functions of the gastric system [2]. Barry Marshall and Robin Warren, who explained the relationship between the bacterium *Helicobacter pylori* and gastric ulcers, won the Nobel Prize in Medicine or Physiology in 2005 [3,4].

Various formulations for oral drug delivery in the treatment of gastric ulcers, such as tablets, capsules and float tablets, have been challenged given their incomplete eradication of *H. pylori*. One reason for the incomplete eradication of *H. pylori* is likely due to the short residence time of drugs in the stomach; thus, effective treatment concentrations cannot be achieved in the gastric mucous layer or epithelial cell surfaces where *H. pylori* exists [5,6]. Another reason might be due to poor stability of drugs or formulations in gastric acid or poor permeability of the drugs across the mucus layer [5,7]. Thus, many researchers have developed several new formulations, such as nanoparticles, mucoadhesive tablets, pH-sensitive excipient composition mucoadhesive microspheres, *etc.*, to increase the time that the drugs reside in the gastrointestinal tract to enhance *H. pylori* eradication [8,9,10,11]. Among these formulations, mucoadhesive microparticles have obtained considerable attention due to their ability to contact with the absorbing mucosa, thereby prolonging the drug’s residence time at or above the site of drug absorption and resulting in an increased concentration gradient that favors drug absorption and localization in specified regions to enhance the drug’s bioavailability [12,13,14].

Over recent decades, a considerable amount of attention has been paid to the utilization of natural polymers for the development of various drug delivery systems owing to their availability, non-toxic properties, cost effectiveness, biodegradability and biocompatibility [15]. Among these polymers, sodium alginate has been investigated widely given its unique characteristic of forming hydrogel beads in the presence of various metal ions, such as Ca^2+^, Ba^2+^, Zn^2+^, Al^3+^, *etc.*, due to an ionic interaction between the carboxylic acid groups located on the polymer backbone and these cations [16,17]. Various drugs have been successfully incorporated in ionotropically cross-linked alginate hydrogels and release profiles depending on their physicochemical properties and method of preparation [18,19,20]. However, the drug release properties of ionotropically cross-linked alginate hydrogels have some disadvantages when used as drug carriers. First, the drug could leak during gel formation due to the long immersion time, which decreased the encapsulation efficiency. Moreover, the burst release of the drug from pure cross-linked alginate microparticles/beads is severe due to the rapid breakdown of beads during the *in vitro* release process [21,22].

**Figure 1:**
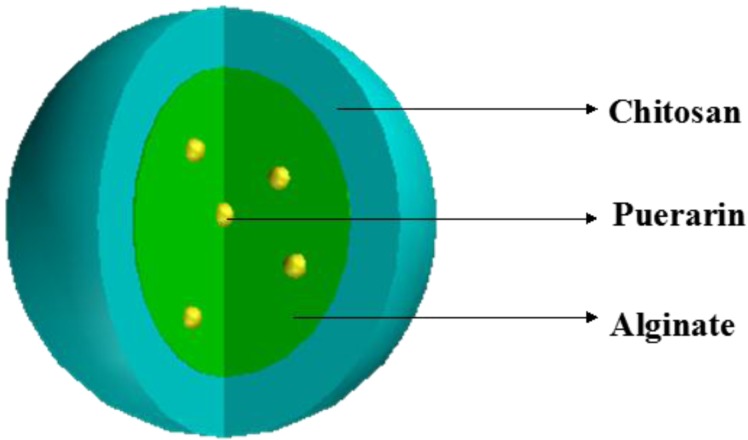
Depiction of a core-shell microparticle containing a drug-loaded Ca-alginate microsphere as the core and chitosan as the outer shell.

Chitosan, a natural polysaccharide [23] derived from chitin by alkaline deacetylation, is one of the most abundant natural polymers on earth and serves as a structural polysaccharide for many phyla of lower plants and animals. Given its suitable biodegradability; biocompatibility; immunological, antibacterial, and wound-healing properties; and its good mechanical and film-forming properties, [24] chitosan has been widely applied in biomedical fields as a hemostat, [25] scaffolds for tissue engineering [26], wound dressing [27], controlled drug [28], and gene [29], delivery vehicle. At the same time, chitosan has been used to reinforce alginate microspheres [30] base on the electrostatic interaction between carboxylate alginate groups and ammonium chitosan groups. The chitosan-alginate complex degrades slowly in phosphate buffer, and this behavior results in suppression of the initial release of drugs occurring for uncoated microspheres [31]. Chitosan is a mucoadhesive polymer with permeation enhancing properties [32]; it facilitates the opening of epithelial tight junctions [4], which prevent sialic acid from eroding mucosa. This may attributed to the electrostatic attraction between its positively charged d-glucose-amine residues and the negatively charged sialic acid residues of mucin. In addition, hydrophobic interactions might contribute to its mucoadhesive properties [33]. As chitosan precipitates at pH values above 6.5, it loses its mucoadhesive and permeability properties. Collectively, these effects reduce the drug’s release in the intestinal tract, thereby making it suitable for gastroretentive delivery. Based on the need for a gastroretentive delivery system as well as the physical and chemical characteristics of chitosan and sodium alginate, we designed a novel core-shell type pH-sensitive mucoadhesive microparticles delivery system. The mucoadhesive microparticles are prepared using a two-stage method, wherein Ca-Alginate (Ca-Alg) gel beads loading puerarin are recovered as a core and subsequently coated with chitosan as the shell (Figure 1), and these techniques are amenable to industrial production.

Puerarin (4′,7-dihdroxy-8-β-glucosylisoflavone), a major isoflavone found in a number of plants and herbs, has been used as a functional food and medicine for a long time. Its comprehensive biological actions have been well-documented by numerous studies, indicating its protective effects in gynecological diseases, diabetic nephropathy, cognitive capability, osteoporosis and cardiovascular diseases [34]. Many researchers have reported that puerarin has a variety of activities, including anti-inflammatory [35], anti-apoptosis [36] and anti-oxidative activities. In addition, puerarin has been administered to treat alcohol-related problems, acting as an anti-intoxication agent [37,38]. It also has exhibited preventive effects on immunological injury [3] and liver damage [39]. Increasing evidence suggests that puerarin can protect gastric mucosa from stress-induced injury [40].

In the present study, we developed a novel core-shell type pH-sensitive mucoadhesive microparticle. Sodium alginate acts as the drug-loaded core beads, and chitosan serves as the coating shell. The complex not only targets puerarin into the gastric mucosa but maintains sustained release. Additionally, we explored the gastric protection and mechanisms of mucoadhesive microparticles loaded with puerarin.

## 2. Experimental Section

### 2.1. Materials and Animals

**Figure 2:**
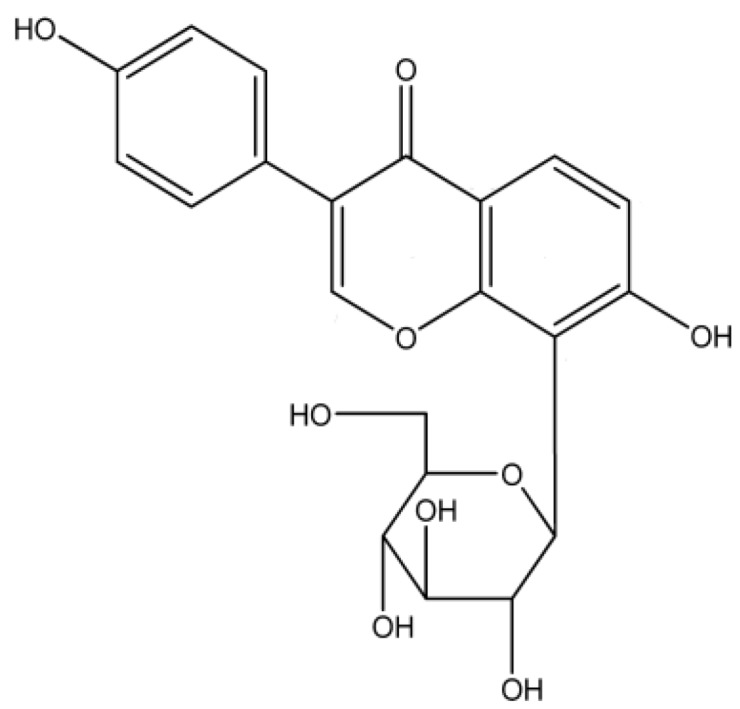
Chemical structure of puerarin.

Puerarin (purity > 99%; molecular structure presented in Figure 2) was obtained from Xi’an Qing Yue Biotechnology Co., Ltd. (Xi’an, Shanxi, China). Sodium alginate, chitosan (with 95% deacetylation and a molecular weight of 650,000) and fluorescein isothiocyanate (FITC) were obtained from Sangon Biotech, Co., Ltd. (Shanghai, China). The omeprazole was purchased from the Youcare Pharmaceutical Group Co., Ltd. (Beijing, China). The rebamipide was obtained from the Otsuka Pharmaceutical Co., Ltd. (Hangzhou, Zhejiang, China). Liquid paraffin was supplied by Beifang Tianyi Chemical Reagent Co. (Tianjin, China). Span-80 and Tween-80 were purchased from Sigma-Aldrich Co. (St. Louis, MO, USA). Micronized CaCO_3_ (40 nm) was provided by Wangyong Technology, Ltd. (Beijing, China). Methanol for HPLC analysis was obtained from Merck Co. (Darmstadt, Germany). Deionized water was obtained from a Milli-Q water purification system (Merck Millipore, Bedford, MA, USA).The simulated gastric fluid (SGF, pH 1.2) composed of 7 mL 36.5% HCl, 2g NaCl and 1000 mL deionized water and the simulated intestinal fluid (SIF, pH 6.8) composed of 6.8g K_2_HPO_4_, 1.2g NaOH and 1000 mL distilled water were prepared as described in Chinese Pharmacopoeia 2010. All other solvents and reagents were analytical grade and used without further modification. The Prostaglandin E_2_ Express EIA Monoclonal Kit was obtained from R&D (Ann Arbor, MI, USA). The IL-6 Mouse ELISA Kit, IL-1β Mouse ELISA Kit and TNF-α Mouse ELISA Kit were obtained from eBioscience (San Diego, CA, USA).

Male Sprague Dawley rats weighing 180–200 g and C57 black mice (6 weeks old, weighing 18–22 g) were obtained from the Vital River Laboratory Animal Technology Co., Ltd. (Beijing, China). The animals were housed under standard conditions of temperature (24 ± 1 °C), relative humidity (55% ± 10%), and 12 h/12 h light/dark cycle and fed standard pellet and water *ad libitum*. Animals were housed for 5 days under these conditions to adapt to the experimental environment. Animals described as fasted were deprived of food for 24 h but had free access to water. All protocols used in this study were in accordance with NIH Guide for the Care and Use of Laboratory Animals and approved by the Ethics Committee of Tianjin University of Traditional Chinese Medicine (TCM-2009-037-E15).

### 2.2. Preparation of Mucoadhesive Microparticles

A modified emulsification-internal gelatin method was employed to prepare Ca-Alg gel beads as described previously [41]. Briefly, 0.20 g of calcium carbonate was added to 20 mL of 1.0% (w/v) sodium alginate solution containing 0.50 g puerarin and placed in an ultrasonic bath for 10 min. The suspension was dispersed into 100 mL of light mineral oil containing 1.0% (v/v) Span 80 and 1.0% (v/v) acetic acid at a stirring speed of 500× *g* at 37 °C for 15 min. The Puerarin-Ca-Alg gel beads were rinsed with 200 mL of 1% (v/v) Tween 80 in aqueous solution and then with 100 mL of deionized water thrice to remove any traces of oil from the Puerarin-Ca-Alg gel bead surface. Puerarin microparticles were prepared by immersing 0.5 mL of Puerarin-Ca-Alg gel beads into a chitosan acetic acid solution (1%, v/v) for 20 min at room temperature. During the coating process, Puerarin-Ca-Alg gel beads were gently shaken to ensure a uniform reaction. Afterwards, Puerarin microparticles were frozen and lyophilized in a Savant ModulyoD freeze-drier (Thermo, Holbrook, NK, USA). The properties of the microparticles are listed in Table 1 (formulations A0-A5). Three batches of microparticles were prepared for further study.

**Table 1:** The mean size of different composition microparticles prepared by emulsification-internal gelatin method.

Formulation	Composition (as(w/w) Ratio)		Coagulation Fluid (%(w/v))	Mean Size (μm) (±S.D.)
	Alginate	Puerarin	Chitosan	
A1	1	0.5	0	50.3 ± 11.2
A2	1	0.5	0.4	69.3 ± 17.6
A3	1	0.5	0.7	86.3 ± 20.5
A4	1	0.5	1	112.3 ± 7.8
A5	1	0.5	1.3	124.7 ± 25.6

### 2.3. Characterization of the Microparticles

#### 2.3.1. Morphological Examination

The morphology of the microparticles was observed via optical microscopy (Nikon FA-35, Tokyo, Japan) and scanning electron microscopy (SEM, Hitachi SU70, Tokyo, Japan). For optical microscopy, the microparticles were directly observed under magnification. Prior to SEM examination, lyophilized microparticles were mounted on mental stubs using double-sided tape and coated with a 150 Å layer of gold under vacuum.

#### 2.3.2. Particle Size Measurement

The particle size of the microparticles was measured using a stage micrometer scale. Dry microparticles (3 mg) were suspended in distilled water and ultrasonicated for 10 s. A drop of suspension was placed on a clean glass slide, and the microparticles were counted under optical microscopy. A minimum of 200 microparticles was counted per batch.

#### 2.3.3. Differential Scanning Calorimetry (DSC) Analysis

DSC was recorded on a Perkin-Elmer DSC7 (Perkin-Elmer, Norwalk, CT, USA). The samples of sodium alginate (A); chitosan (B); puerarin (C); blank microparticles (D) and puerarin microparticles (E) were freeze-dried. Lyophilized samples (5–8 mg) were heated from 30 to 350 °C in crimped standard aluminum hermetic pans at a heating rate of 10 °C per min with a constant purging of nitrogen at 100 mL per min. The system was calibrated with indium (melting point of 156.86 °C).

#### 2.3.4. Fourier Transform Infrared Spectroscopy (FT-IR)

The FT-IR transmission spectra were recorded on a Bio-Rad FTS 3000 spectrophotometer (Bio-Rad, Richmond, CA, USA) in the wave number range 4000–400 cm^−1^ using KBr pellets. The samples of sodium alginate (A); chitosan (B); puerarin (C); blank microparticles (D) and puerarin microparticles (E) were freeze-dried, and a total of 2% (w/w) of samples were mixed with potassium bromide. The mixtures were ground into fine powders, and disks were compressed for scanning. Each sample was assessed in triplicate at a minimum.

### 2.4. Determination of Drug Loading, Encapsulation Efficiency and Swelling Ratio

Drug loading and encapsulation efficiency were measured after extracting drug from the prepared puerarin microparticles. Briefly, 100 mg of drug-loaded microparticles were added to 10 mL of methanol and sonicated for 30 min to ensure the complete extraction of puerarin. Each test group was performed in triplicate. After equilibrium was attained, the samples were filtered through 0.22-μm pore size syringe filters and assayed by HPLC. Determination was performed on a Waters 2695 system (Waters, New York, NY, USA) equipped with a 2487 dual λ absorbance detector. 5 μL of samples were injected onto a Thermo Hypersil ODS-2 Column (250 mm × 4.6 mm, 5 μm) at 25 °C. All samples were detected with a mobile phase of a mixture of methanol-water (75:25, v/v) at a flow rate of 1.0 mL/min. Chromatograms were monitored at 250 nm. The drug loading (%) and encapsulation efficiency (%) were calculated using the following equations, respectively:

(1) $$\text{drug loading(\%)}=\frac{\text{weight of drug in sample}}{\text{weight of sample}}\times 100$$

(2) $$\text{Encapsulation efficiency(\%)}=\frac{\text{wegiht of drug in sample}}{\text{theoretical drug loading}}$$

The swelling properties of puerarin microparticles were assessed in the SGF (pH 1.2). Puerarin microparticles (0.10 g) were placed in the baskets of intelligent dissolution apparatus (ZRS-8G, Tianjin University Wireless factory, Tianjin, China) containing 200 mL of SGF (pH 1.2) and stirred at 37 ± 0.5 °C at 50 rpm. The swelled beads were removed at predetermined time intervals and weighed after drying the surface with tissue paper. The swelling ratio was determined using following formula:

(3) $$Swelling\;ration=\frac{Weight\;of\;beads\;after\;swelling-Dry\;weight\;of\;beads}{Dry\;weight\;of\;beads}\times 100$$

### 2.5. Drug Release Study in Vitro

*In vitro* release experiments were performed using the USP 30 NO. 2 dissolution test apparatus fixed with six rotating paddles (ZRS-8G, Tianjin University Wireless Factory, Tianjin, China). The release media are SGF (pH 1.2) and SIF (pH 6.8) (500 mL, 37.5 ± 0.5 °C), and the rotation speed was 50 ± 1 rpm. The dried microparticles (500 mg) were placed into each cup, and 5 mL of solution was withdrawn from the release medium at regular intervals, and the same amount of the fresh buffer solution was added to maintain a constant volume. The collected solution (2 mL) was filtered through a membrane with a pore size of 0.22 μm. Each test group was performed in triplicate. The concentration of puerarin was assayed using the above mentioned HPLC method, and then the cumulative percentage of puerarin released was obtained. The dissolution results were the average of three measurements.

### 2.6. Evaluation of Mucoadhesiveness in Vitro

The puerarin microparticles were tested for mucoadhesiveness according to the method designed by Rao and Buri [42]. Briefly, the stomachs obtained from male Sprague-Dawley rats (180–200 g) subjected to fasting 24 h were opened along the great curvature and rinsed in SGF (pH 1.2). In addition, the small intestines (jejunum) obtained from the same rats were cut longitudinally and rinsed in physiological saline. The experiment to evaluate the adhesive properties began within 2 h after dissection. Stomach tissue was cut into 2 × 1 cm pieces, and jejunum tissue (4 cm in length) was prepared. Two-hundred microspheres of each were scattered uniformly on the surface of the stomach mucosa. Then, the mucosa with the microparticles was placed in a chamber maintained at 93% relative humidity and room temperature. After 20 min, the tissues were removed and fixed on a polyethylene support at a 45° angle. The stomach and intestine tissues were rinsed with SGF (pH 1.2) and SIF (pH 6.8) for 5 min at a rate of 22 mL/min. The microparticles remaining at the surface of the gastric mucosa were counted, and the percentage of the remaining microparticles was calculated and statistically analyzed.

### 2.7. Fluorescence Imaging of the Gastrointestinal Tract 

To observe the behavior of microparticles *in vivo* more precisely, chitosan was labeled with FITC via chemical reaction at the isothiocyanate group of FITC and the primary amino group of chitosan [43]. In brief, FITC-labeled chitosan was synthesized by adding 10 mL of methanol followed by 5 mL of FITC in methanol (2.0 mg/mL) to 10 mL of chitosan (1% in 0.1 M CH_3_COOH) in the dark at ambient temperature. After 5 h, the labeled polymer was precipitated in 0.2 M NaOH. The precipitate was pelleted at 35,000× *g* (15 min) and washed with methanol: water (70:30, v/v). The washing and pelletization were repeated until no fluorescence was detected in the supernatant (Perkin-Elmer LS-5B Luminescence spectrometer, Beaconsfield, England, λ_exc_ = 490 nm, λ_emi_ = 520 nm). Finally, the labeled chitosan was frozen and lyophilized.

C57 black mice were fasted overnight with free access to water before the experiments. A certain amount of FITC-labeled chitosan (FITC-CS)-coated microparticles was administered into fifteen male C57 black mice. At certain times (0, 2, 4, 6, and 8 h), the mice were sacrificed. Then, the stomach, duodenum, jejunum and colon were excised and observed using the Kodak In-vivo Imaging System FX Pro (Kodak, Rochester, NY, USA).

### 2.8. Gastro-Protective Studies in Rats

#### 2.8.1. Ethanol-Induced Gastric Injury in Rats

The rats were deprived of food but had *ad libitum* access to tap water for overnight before ulcer induction. Gastric mucosal damage was induced in conscious rats by gavage of 5.0 mL/kg of absolute ethanol. Puerarin microparticles were suspended in normal saline. Animals were randomized into eight groups (*n* = 6): control, ethanol, omeprazole (20 mg/kg) + ethanol, rebamipide (100 mg/kg) + ethanol, puerarin microparticles (150 mg/kg) + ethanol, puerarin microparticles (300 mg/kg) + ethanol, puerarin microparticles (450 mg/kg) + ethanol, and puerarin microparticles (600 mg/kg) + ethanol. All of the rats in groups were pre-treated by gavages with puerarin microparticles and control drugs (omeprazole and rebamipide) 120 min prior to ethanol administration. Four hours later, the animals were sacrificed, and their stomachs were excised. Each stomach was cut along the greater curvature and rinsed thoroughly with normal saline; then, macroscopic determination of the gastric mucosal injury index was performed.

#### 2.8.2. Determination of the Ulcer Index and Percent Inhibition

The ulcer index (UI) and percent inhibition were calculated in ethanol-induced rats. To determine the ulcer index, the stomach was examined for ulceration using a simple dissecting microscope. The stomach was examined under the microscope to observe erosions. The stomachs were scored using the following scale: 1, small round hemorrhagic erosion; 2, hemorrhagic erosions <1 mm; 3, hemorrhagic erosion 2–3 mm in size; and 4, hemorrhagic erosion >4 mm. The scores were multiplied by 2 when the width of the erosion was larger than 1 mm [44].The UI for each animal was calculated as the mean ulcer score (mm^2^). The percentage of inhibition (I %) was calculated using the following formula:

(4) $$I\%=(\frac{{U\;A}_{\;control}-{U\;A}_{\;treated}}{{U\;A}_{\;control}})\times 100$$

#### 2.8.3. Histopathology

A small fragment of the gastric wall from each animal was fixed in 10% buffered formalin solution followed by tissue dehydrated with alcohol and xylene. Then, each sample was embedded in paraffin wax and sectioned into 5-μm slides prior to staining. To evaluate mucus production, periodic acid Schiff Base (PAS) was used for initiative staining following the manufacturer’s instructions (Periodic Acid-Schiff (PAS) Kit, Sigma-Aldrich Co., St. Louis, MO, USA). Then, some slides were also stained with hematoxylin and eosin (H & E).

#### 2.8.4. Measurement of Cytokine, Production (TNF-α, 1L-1β, IL-6 and PGE_2_)

A 10% (v/w) gastric mucosa homogenate was prepared in PBS buffer (10 mM phosphate buffer, pH 7.4, 130 mM NaCl, and 4 mM KCl) and centrifuged at 12,000× *g* for 10 min at 4 °C. The resultant supernatant was used to evaluate TNF-α, IL-6, 1L-1β and PGE_2_ levels in rat stomach tissues using corresponding ELISA kits according to the manufacturers’ instructions. Total protein concentrations in the supernatants were determined using a Pierce BCA Protein Assay Kit (Rockford, IL, USA). Furthermore, the relative amounts of TNF-α, IL-6, 1L-1β and PEG_2_ were determined as milligram of total protein in the gastric mucosa tissues.

### 2.9. Statistical Analysis

The results analysis for the *in vitro* acitvity of puerarin microparticles were expressed as means ± S.D. and the results of *in vivo* activity of puerarin micropartivles were expressed as means ± S.E. The statistical analysis was performed by analysis of variance (ANOVA) followed by Dunnett’s test. The data were evaluated with SPSS 17.0 (SPSS Inc., Chicago, IL, USA). The criterion for statistical significance was *p <* 0.01 or *p <* 0.05.

## 3. Results and Discussion

### 3.1. Characterization of the Puerarin Microparticles

#### 3.1.1. Morphological Observations of Puerarin Microparticles

**Figure 3:**
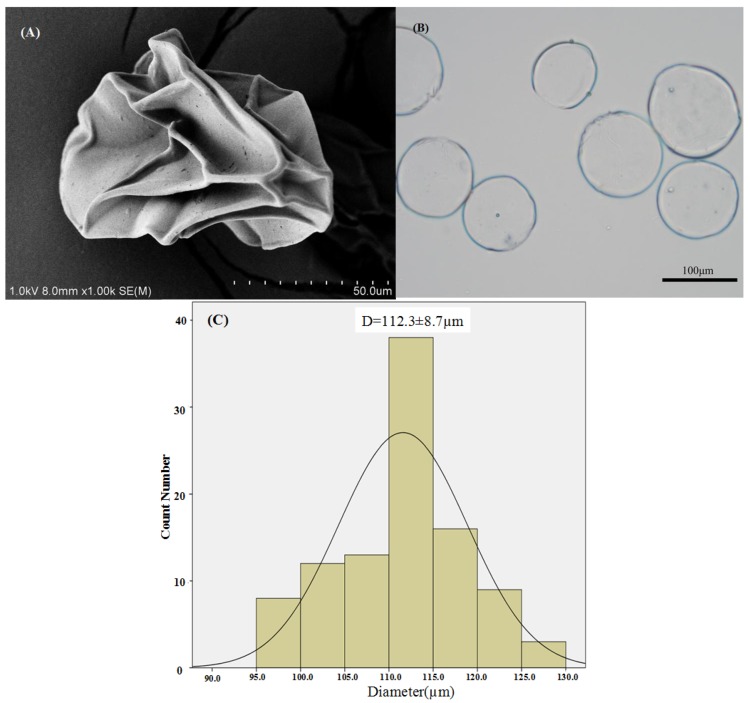
The morphological characterization of the puerarin microparticles. The SEM micrograph of puerarin microparticles (**A**); optical micrograph of puerarin microparticles (**B**); and particle size distribution of puerarin microparticles (**C**).

The morphological characterization of the puerarin microparticles was performed by optical microscopy and SEM (Figure 3). As shown in Figure 3B, microparticles prepared by this method appeared as well-rounded spheres with uniform size distribution under optical microscopy. This arrangement was attributed to the fact that the alginate gel beads were formed from the interaction between Ca^2+^ and guluronic acid residues of alginate, and puerarin microparticles were obtained by chitosan solidification. Figure 3A indicates that after lyophilization, the puerarin microparticles exhibited very rough surfaces with characteristic large wrinkles. This phenomenon may be caused by the unique structure of the Ca-Alg gel, which contains 99%–99.5% water [45]. Interestingly, the coarse and wrinkled microparticle surface might improve its mucoadhesion *in vivo* [46]*.*

#### 3.1.2. Particle Size

Previous reports suggested that the size of the beads increases with increasing concentrations of chitosan in the coagulation fluid [2]. In this study, the mean particle size of the microparticles (formulations A0–A5) increased from 50.3 to 124.7 μm as the concentration of chitosan increased in the coagulation fluid (Table 1). Furthermore, Figure 3C indicates a narrow particle size distribution of microspheres.

#### 3.1.3. DSC analysis

**Figure 4:**
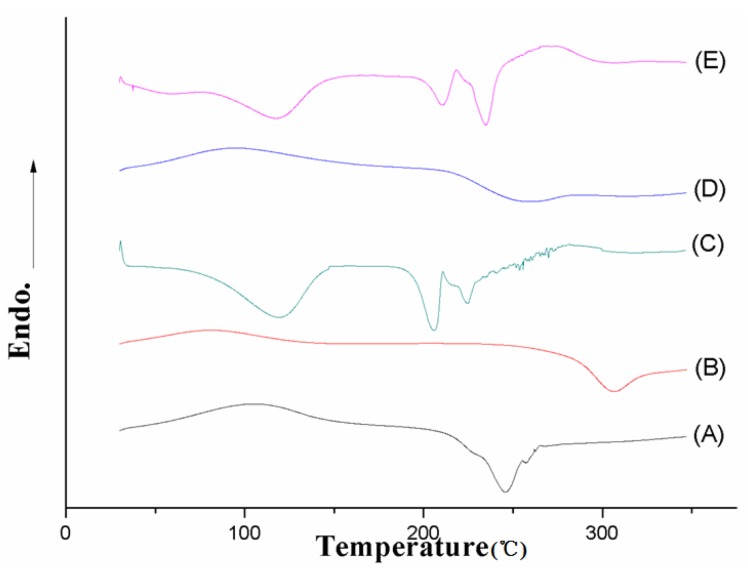
DSC thermograms of sodium alginate (**A**); chitosan (**B**); puerarin (**C**); blank microparticles (**D**) and puerarin microparticles (**E**).

The DSC characteristics of sodium alginate (A); chitosan (B); puerarin (C); blank microparticles (D) and puerarin microparticles (E) are presented. All of the curves were merged into one picture after standardizing the units and scales. In the calorimetric studies, the thermogram of sodium alginate is characterized by an exothermic peak at 246.1 °C due to polymer degradation and exhibits the temperature for sodium alginate degradation (Figure 4A). The DSC scans of chitosan demonstrate an exothermic baseline deviation at approximately 305 °C, which indicates the onset of chitosan degradation (Figure 4B). The puerarin powder exhibited a sharp but wide endothermic peak at 234.56 °C and 207.11 °C, indicating the melting points and that puerarin was in the polymorphs. However, Figure 4D indicates that the spectrum of puerarin microparticles exhibited neither a single nor a double characteristic exothermic peak of two polymers but rather a slightly broad band at a range of 222–277 °C, which indicates the interaction between chitosan and alginate in the blank microparticles. As shown in Figure 4E, the melting point of puerarin microparticles was slightly changed from 217.2 °C to 240.4 °C. The shapes of the peaks were also altered as the peak at 217.2 °C was reduced and the peak at 240.2 °C was stronger.

#### 3.1.4. FT-IR Spectroscopy

**Figure 5:**
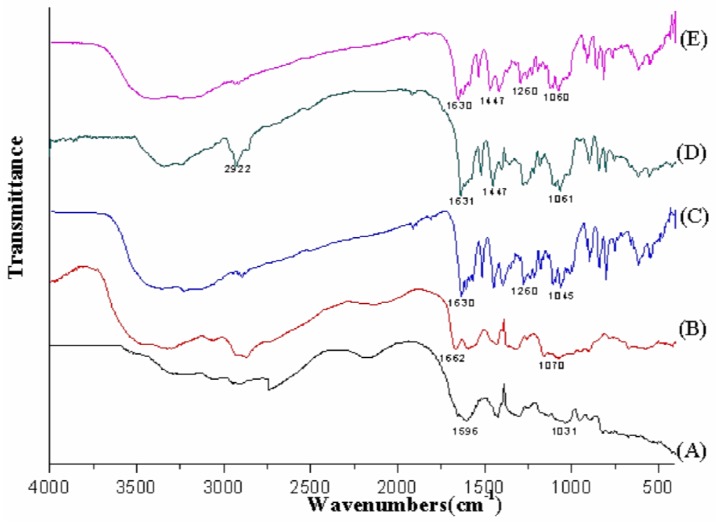
The FT-IR spectra of different compounds of puerarin microparticles. FT-IR spectra of sodium alginate (**A**); chitosan (**B**); puerarin (**C**); blank microparticles (**D**) and puerarin microparticles (**E**).

FT-IR analysis allows for the observation of vibration modes for the specific groups of compounds analyzed [47]. Therefore, infrared spectra of sodium alginate (A); chitosan (B); puerarin (C); blank microparticles (D) and puerarin microparticles (E) are presented in Figure 5. In the sodium alginate IR spectrum (Figure 5A), we observed vibration mode in the band of 1596 to 1421 cm^−1^, which can be attributed to carboxyl group (COO-). A wide absorption band around 1031 cm^−1^ was observed due to the C-OH stretch. Moreover, the IR spectrum of chitosan (Figure 5B) exhibits peaks at 1662, 1590, and 1428 cm^−1^, characterizing the amino groups (-NH^3+^); a band around 1070 cm^−1^ can be attributed to –CH-OH stretch. As shown in Figure 5C, there are three absorption peaks at 1630, 1588 and 1260 cm^−1^, which correspond to the aldehyde group (C=O) stretching vibration, benzene skeleton vibration, and C-O absorption peak. By analyzing the IR spectra of the absorption spectrum of the blank microparticles (Figure 5D), differences were noted in the vibration modes of their peaks compared with the polymers alone. In detail, the appearance of the bands at 1631 cm^−1^, 1447 cm^−1^ and 2922 cm^−1^ as well as the disappearance of the band at 1590 cm^−1^ (the characteristic peak of the chitosan amino group) suggest the formation of a polyelectrolyte complex between sodium alginate and chitosan. As shown in Figure 5E, two wide absorption band around 1447 and 1060 cm^−1^ are noted that are similar to the infrared spectrum of the blank microparticles, indicating a polyelectrolyte complex has been formed. In addition, two characteristic peaks of puerarin at 1630 and 1260 cm^−1^ in the infrared spectra of puerarin microparticles are evident, which indicates that puerarin has been loaded into the microparticles.

### 3.2. Drug Loading, Encapsulation Efficiency and Swelling 

Variation in the concentrations of chitosan greatly impacts drug loading and encapsulation efficiency in chitosan-alginate beads. In the absence of chitosan, the drug loading of microparticles was very low (5.1%, Table 2, A1), which is potentially attributed to insufficient cross-linking and the fact that the structure of the gel network allows puerarin diffusion. The addition of 0.4%–1.0% of chitosan to the coagulation fluid (A2, A3, and A4) resulted in a large increase of the drug loading and encapsulation efficiency owing to increased firmness in the alginate-chitosan complex between the carboxylate groups in the alginate and the protonated amine groups in the chitosan. As a result, less puerarin was lost during gelation. In the presence of more chitosan, this process also occurs due to the increased number of alginate-chitosan ionic linkages. However, the drug loading may be relatively low than others, which may be attributed to the multilayer chitosan microparticles (A5).

**Table 2:** The entrapment and swelling ratio of different compositions microparticles prepared by emulsification-internal gelatin method.

Formulation	Entrapment Efficiency (%)	Drug Loading (%)	Swelling Ratio (%) (±S.D.)
A1	70.3	5.1	69 ± 11
A2	84.3	10.4	132 ± 18
A3	90.8	15.9	179 ± 12
A4	98.5	34.7	222 ± 2
A5	99.2	23.2	175 ± 11

Previous articles reported that the adhesive properties and cohesiveness of mucoadhesive polymers are generally affected by their swelling behavior [48]. Mucoadhesive microparticles take up water from the underlying mucosal tissue by absorbing, swelling, and capillary effects, leading to considerably stronger adhesion [49]. Alginate beads coated with chitosan exhibited increased swelling ability (132%–222%), with an optimal chitosan concentration of 1% (w/v) with 222% swelling ratio that was reduced to 179% with 0.7% (w/v) chitosan. The lower chitosan concentration may be insufficient to protect air bubbles inside the beads from the surrounding medium, resulting in shrinking. Higher (1.3% (w/v)) chitosan exhibited a swelling ratio comparable to 0.7% (w/v) chitosan owing to an increased concentration of coagulation fluid, which yielded larger, thicker beads. The thicker chitosan layer may cause solution uptake, leading to a higher bulk density than the external medium and shrinking [50]. Based on the drug loading, encapsulation efficiency and swelling ratio data, 1% chitosan was chosen as the optimal coagulation fluid.

### 3.3. In Vitro Drug Release Results

**Figure 6:**
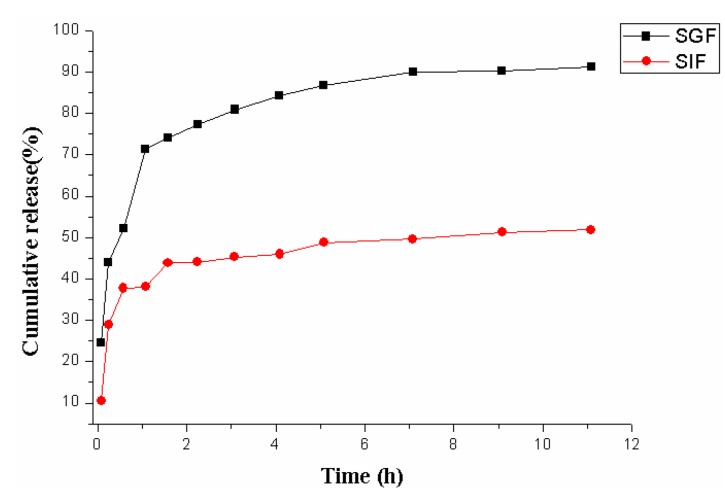
Puerarin release curves in different media: SGF (pH 1.2) and SIF (pH 6.8).

The *in vitro* drug release studies were performed for puerarin mucoadhesive microparticles composite beads in SGF (pH 1.2) and SIF (pH 6.8) (500 mL, 37.5 ± 0.5 °C) for 12 h, respectively. As shown in Figure 6, the puerarin release profile from microparticles in SGF exhibited different behaviors compare with that in SIF. The time taken to release stable in SGF and SIF was 7 ± 0.2, 5 ± 0.3 h, respectively. After 12 h, the cumulative release of the puerarin microparticles was 93.50% in SGF, considerably higher than that in SIF (52.18%). The increased release of puerarin from microparticles might be attributed to the increased solubility of chitosan in acidic medium. Thus, from a more practical point of view, the puerarin mucoadhesive microparticles can liberate substantial amounts of puerarin in acidic gastric fluid and minimize the release in the intestines, achieving the purpose of the controlled release of puerarin side-specific particles in the stomach.

### 3.4. In Vitro Evaluation of Mucoadhesiveness

The *in vitro* adhesiveness test (Table 3) revealed that the percentage of puerarin microparticles remaining on the gastric mucosa (92.6% ± 1.7%, *n* = 3) was increased compare with the small intestinal mucosa (30.6% ± 1.7%, *n* = 3). The increased mucoadhesion of puerarin microparticles was expected to be directly related to the pH of the medium as demonstrated with chitosan. Therefore, results suggested microparticles possessed a better mucoadhesivity in stomach.

**Table 3:** Percent of Microparticles Remaining after Rinsing Mucosa of the Stomach and Small Intestine with SGF and SIF, respectively.

Number	% of Microparticles Remaining	
	Stomach SGF	Small Intestine SIF
1	92.5	30.5
2	91.0	29.0
3	94.5	32.5
Average	92.6 ± 1.7	30.6 ± 1.7

### 3.5. Fluorescence Imaging of the Gastrointestinal Tract

**Figure 7:**
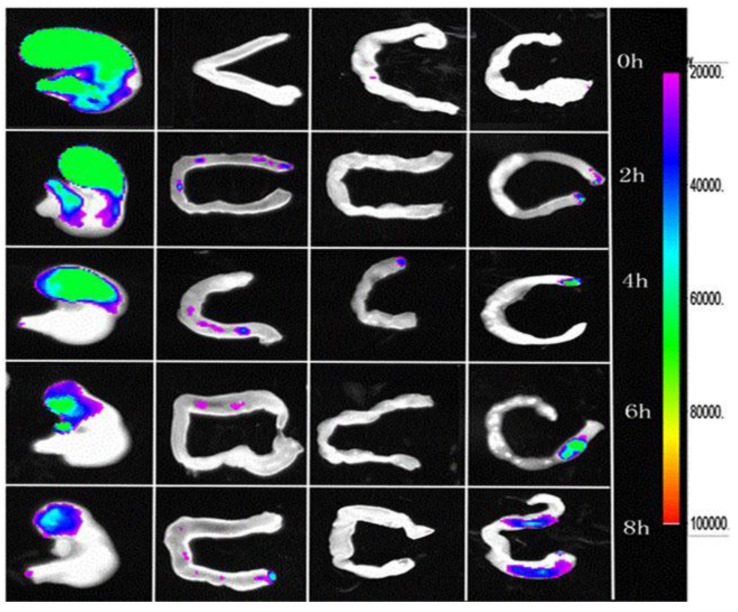
The fluorescence intensity of FITC-CS microparticles in different tissues (Left→Right: Stomach, Duodenum, Jejunum and Colon) varied from 0 to 8 h.

We assumed that increasing gastric residence time of the microparticles is one of the key features that enhances the bioavailability of puerarin in the gastric mucosa. The gastric residence time of FITC-CS microparticles were monitored using the *In-vivo* Imaging System FX Pro (Kodak, Rochester, NY, USA) as shown in Figure 7. These results suggested that FITC-CS microparticles adhere to the gastric mucosa after intragastric administration. Even at 6 h and 8 h, the fluorescence intensities of FITC-CS microparticles on the surface of gastric mucosa were still relatively high, which indicated that the microparticles possess enhanced mucoadhesive properties; therefore, microparticles adhered to the gastric mucosa prevent gastric acid from eroding the gastric mucosa. This phenomenon may be explained by the interaction between the carboxy groups of chitosan and the gastric acid in mucin molecules, which is responsible for enhancing the permeability of gastroretentive delivery and enhancing the effective drug absorption [33,51].

### 3.6. Gastro-Protective Study Results

#### 3.6.1. Ulcer Index and Percent Inhibition 

**Figure 8:**
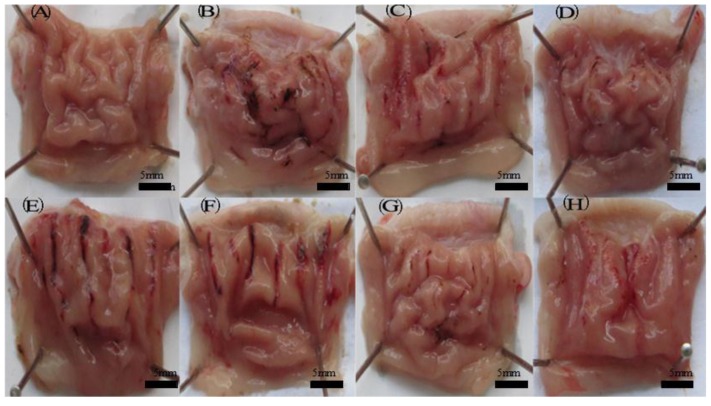
Effect of puerarin microparticles on ethanol-induced gastric ulcer in rats. (**A**) Control group; (**B**) Ethanol group, 5.0 mL/kg; (**C**) Omprazole, 20 mg/kg; (**D**) Rebamipide, 100 mg/kg; (**E**) Puerarin microparticles, 150 mg/kg; (**F**) Puerarin microparticles, 300 mg/kg; (**G**) Puerarin microparticles, 450 mg/kg; (**H**) Puerarin microparticles, 600 mg/kg. All drugs were orally administered.

The anti-ulcer effects of puerarin mucoadhesive microparticles on the ethanol-induced ulcer model in rats are presented in Figure 8. When rats were treated with ethanol (5 mL/kg) alone, extensive and visible gastric lesions were observed in the surface epithelial cells of gastric mucosa (Figure 8B) with an UI score of 24.72 ± 1.32. However, pre-treatment with puerarin mucoadhesive microparticles protected ethanol-induced gastric injury in a dose-dependent manner (Table 4). At a dose of 600 mg/kg, puerarin mucoadhesive microparticles significantly (*p* < 0.01) reduced the intensity of gastric mucosal damage and demonstrated increased gastroprotective activity (79.3%) compared with the control drug rebamipide (77.3%). The results of the present study demonstrated that animals pretreated with puerarin mucoadhesive microparticles (Figure 8E–H) exhibited a dose-dependent protection of mucosal layer damage and a substantial reduction in continuous gastric mucosa hyperemia (Figure 8A). Ethanol consumption is known to be one of many factors causing gastric ulcer formation owing to the generation of oxygen-derived free radicals, such as superoxide anions, lipid peroxides, and hydroxyl radicals [52]. The obtained results indicate that the puerarin mucoadhesive microparticles may exhibit strong antioxidant and radical scavenging potential, suppressing ethanol-induced depletion of gastric mucosa in rats.

**Table 4:** The effect of puerarin microspheres on gastric ulcer index and inhibition of ethanol-induced gastric mucosal lesions in rats.

Animal Group	Pre-Treatment	Ulcer Index (Score) (Mean ± S.E.)	Inhibition (%)
A	Normal control	--	--
B	Ulcer control	24.72 ± 1.32	0
C	Omeprazole (20 mg/kg)	4.93 ± 0.36	80.0
D	Rebamipide (100 mg/kg)	5.61 ± 0.55	77.3
E	Microspheres (150 mg/kg)	19.96 ± 1.15	19.3
F	Microspheres (300 mg/kg)	12.53 ± 0.98	49.3
G	Microspheres (450 mg/kg)	7.92 ± 0.76	68.0
H	Microspheres (600 mg/kg)	5.87 ± 0.61	76.2

#### 3.6.2. Histological Results

**Figure 9:**
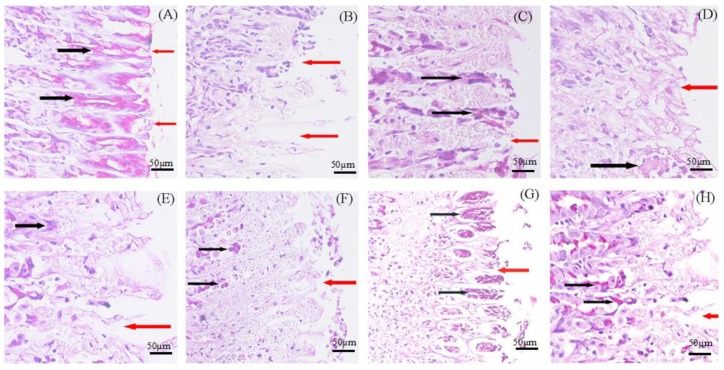
Effect of puerarin microparticles on gastric tissue glycoprotein-PAS staining of ethanol-induced gastric damage in rats. (**A**) Control group; (**B**) Ethanol group; 5 mL/kg; (**C**) Omprazole, 20 mg/kg; (**D**) Rebamipide, 100 mg/kg; (**E**) Puerarin microparticles, 150 mg/kg; (**F**) Puerarin microparticles, 300 mg/kg; (**G**) Puerarin microparticles, 450 mg/kg; and (**H**) Puerarin microparticles, 600 mg/kg. The black arrows indicate the glycoprotein, which appears as a magenta stain, and the red arrows indicate loss of continuity. (PAS stain 200×).

To confirm the results of preventing ethanol-induced gastric damage, the stomachs were evaluated by histological observation using PAS staining in the superficial layer of the gastric mucosa. The PAS staining abnormality is used to observe glycogen levels in tissues. The gastric mucosa in animals pretreated with standard drugs (omeprazole and rebamipide) (Figure 9C,D) or puerarin mucoadhesive microparticles (Figure 9E–H) exhibited substantial expansion of a continuous PAS-positive mucous gel layer that lined the entire gastric mucosal surface (magenta color), indicating an increase in the glycoprotein content of the gastric mucosa in pretreated rats. As shown in Figure 9, rats treated with absolute ethanol exhibited disrupted epithelial cells from the upper portion of fundic glands, vacuolization, necrosis and deep dilated inter-glandular spices (Figure 9B) compared with the stomach of normal animals (Figure 9A). Pre-treatment with puerarin mucoadhesive microparticles (Figure 9E–H) or standard drugs (omeprazole and rebamipide) (Figure 9C,D) afford relatively enhanced protection by decreasing gastric ulcer damage, with only slight damage observed in the superficial epithelium (Figure 9G–H). Puerarin microparticles (Figure 9H) exhibited slightly enhanced reduction in gastric ulcer damage compared with the standard drug rebamipide (Figure 9D). With regard to reducing epithelial cell damage, puerarin microparticles protected against mucosal layer damage in a dose-dependent fashion.

#### 3.6.3. Measurement of TNF-α, 1L-1β, IL-6 Expression and PGE_2_ Production

**Figure 10:**
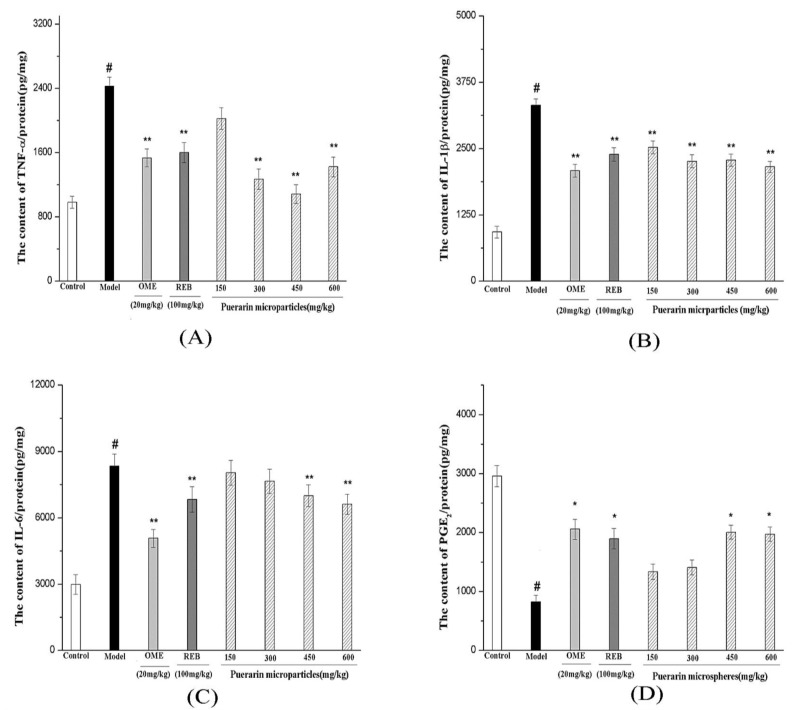
Effect of puerarin microparticles on TNF-α, IL-6, IL-1β, and PGE_2_ levels in ethanol-induced gastric mucosa tissues. TNF-α (A); IL-1β (B); IL-6 (C) and PGE_2_ (D) in stomach tissue were analyzed by commercially available ELISA kits. # *p* < 0.05 (*n* = 6) compared with the control group, * *p* < 0.05; ** *p* < 0.01 (*n* = 6) compared with the ethanol group.

Excessive alcohol consumption can produce acute hemorrhagic gastric erosions, and even result in gastritis characterized by mucosal edema, sub-epithelial hemorrhages, cellular exfoliation, and inflammatory cell infiltration, which is an essential acute inflammatory reaction [53]. TNF-α, 1L-1β, IL-6 and PGE2, which are related to the immunopathology of acute or chronic inflammatory diseases, were considered as primary pro-inflammatory cytokines and mediators. As Figure 10 demonstrates, ethanol-induced gastric injury resulted in a burst of TNF-α (A); 1L-1β (B); IL-6 (C) expression and PGE_2_ (D) production, whereas mice administered standard drugs (omeprazole and rebamipide) and puerarin mucoadhesive microparticles (300, 450 and 600 mg/kg) exhibited significantly reduced increased in TNF-α, 1L-1β and IL-6 (*p <* 0.01) and antagonized the reduction of PGE_2_ in a concentration-dependent manner.

Considering the industrial process and the possibility of clinical application in the future, we designed a novel core-shell type pH-sensitive mucoadhesive microparticle containing puerarin. This novel mucoadhesive microparticle uses a unique biomaterial combination of chitosan and sodium alginates, which are FDA approved and successfully used in a variety of biomedical applications and products. To improve the mucoadhesive properties of polymeric excipients, numerous attempts have been undertaken based on the gastric-specific delivery, such as thiolated chitosans [6] and chitosan–carboxymethylcellulose [54]; however, these matrix materials do not have FDA approval and have not been used in clinical applications. However, we design this gastric-specific delivery system using FDA-approved materials and simple technological preparations, thereby potentially allowing the realization of industrial production.

Peptic ulcers arise from imbalances between offensive factors, such as gastric acid, and protective factors, such as prostaglandins. Gastric acid plays an important role in the pathogenesis of gastric ulcers as stated by the dictum of Karl Schwartz—“no acid, no ulcer” [55]—which has been quoted for decades. Over the last century, therapies for peptic ulcers have evolved from surgery to acid suppressants along with *Helicobacter pylori* eradication [8]. Omeprazole, a benzimidazole proton pump inhibitor (H^+^/K^+^ATP-ase), has anti-inflammatory and antioxidant properties in addition to its ability to stimulate gastric mucus secretion [56]; this agent serves as one of the most effective ulcer treatment to date. Rebamipide protects the gastric mucosa and promotes the quality of ulcer healing by increasing the glycoprotein content in gastric mucus and decreasing reactive oxygen species [57]. Regarding the treatment of gastric ulcers, both omeprazole and rebamipide rely on a single or partial mechanism, which is not perfect. The developed preparation combines above-standard drug advantages. First, the volume of mucoadhesive microparticles is relatively small, whereas the specific surface area is relatively large. After administration, microparticles quickly form a layer of membrane on the surface of the gastric mucosa, protecting the gastric mucosa from erosion induced by a variety of agents, including ethanol and gastric acid. Moreover, puerarin is the main isoflavone glycoside and a major active ingredient extracted from the traditional Chinese medicine Radix Puerariae. Its many biological actions have been reported by numerous studies, including inflammation [35] and oxidation effects [58] as well as the effects on vascular smooth muscle cells [59] and endothelial cells, [60] which suggest that this compound is an effective medicine used to treat gastric ulcers. More importantly, the developed mucoadhesive microparticles increase the residence time of drugs and sustained drug release for several hours in the stomach so effective drug concentration of drugs can be achieved at the surface of the gastric mucous, eradicating *H. pylori* completely.

These puerarin mucoadhesive microparticles exhibited pH-sensitive properties and extended the drug retention time in the stomach during *in vitro* and *in vivo* evaluations. These results were attributed to the use of sodium alginate as the core bead and chitosan as the coating shell. We determined the antiulcer action of puerarin mucoadhesive microparticles using a rat model of ethanol-induced gastric ulcer. This model is commonly used to evaluate the activity of antiulcer agents. A previous study demonstrated that ethanol-induced gastritis influences the formation of experimentally induced gastric injury in rats [61]. We demonstrated the protective action of puerarin mucoadhesive microparticles against ethanol-induced gastric injury through anti-inflammatory actions accomplished via reduced levels of cytokines, such as TNF-α, 1L-1β, IL-6 and PGE_2_. Puerarin mucoadhesive microparticles serve as an effective approach for targeting drug release at its site of absorption, sustaining its release, improving its oral availability and promoting gastro-protective effects in the treatment of gastric ulcers.

## 4. Conclusions

In this work, pH-responsive mucoadhesive microparticles of puerarin with a core-shell structure were successfully prepared using an emulsification-internal gelatin method. The encapsulation efficiency of the optimized microparticles containing puerarin was increased compared with other agents. The *in vitro* release test and mucoadhesive tests suggest that the mucoadhesion and cumulative release of puerarin mucoadhesive microparticles were influenced by the pH of the test medium. Based on fluorescence imaging of the gastrointestinal tract, the puerarin mucoadhesive microparticles were retained in the gastrointestinal tract for an extended period of time, adhering to the surface of the gastric wall and improving puerarin bioavailability at the gastric mucosa. Puerarin mucoadhesive microparticles were clearly demonstrated to function as an antiulcer agent by enhancing the gastric mucosal defense. In the puerarin mucoadhesive microparticles-pretreated groups, the microparticles reversed the decrease in PAS staining induced by ethanol; significant increased TNF-α, IL-1β and IL-6 levels; and decreased the level of PGE_2_.
